# Explainable active reinforcement deep learning improves lung cancer detection from CT images

**DOI:** 10.1038/s41598-026-38239-7

**Published:** 2026-02-24

**Authors:** Ghada Nady, Ahmed Salem, Osama Badawy, Samia Abo-ElNour

**Affiliations:** 1 College of Computing and Information Technology, Arab Academy for Science, Technology and Maritime Transport (AASTMT), Alexandria, Egypt; 2 College of Computing and Information Technology, Arab Academy for Science, Technology and Maritime Transport (AASTMT), Cairo, Egypt; 3 Consultant Diagnostic and Interventional Radiologist in College of Medicine, Ain Shams University, Cairo, Egypt

**Keywords:** Lung cancer detection, CT image, Active reinforcement deep learning, Explainable AI, Attention fusion feature, Feature engineering, Cancer, Computational biology and bioinformatics, Engineering, Mathematics and computing

## Abstract

Lung cancer remains a global health challenge that requires early and accurate diagnosis through medical imaging analysis. This study introduces ARXAF-Net framework which integrates Active Reinforcement deep leaning with strategic feature engineering, selection, advanced classification techniques with Explainable AI. Firstly, The ARXAF-Net framework overcomes the challenges of labeled dataset limitation by leveraging active reinforcement learning where the model achieves a remarkable 99.0% training accuracy using reinforcement learning. After that, Traditional feature extraction techniques, including GLCM and LBP, are combined with CNN with attention fusion model features, forming comprehensive vectors. Advanced techniques pinpoint essential characteristics for classification. The experiments conducted on a dataset comprising 30,020 CT images categorized into two classes—15,010 non-cancer and 15,010 cancer—demonstrate that CNN models employing attention fusion with traditional feature extraction achieve remarkable consistency, reaching a testing accuracy of 99%. Basic CNN models with traditional feature extraction, following normalization, display commendable performance, nearing an accuracy of 95%. Additionally, integrating explainable AI (XAI) into the performance frameworks significantly enhances the outcomes by incorporating feedback from radiologists. This research offers valuable insights into the optimal combinations of preprocessing, feature engineering/selection, and classification algorithms aimed at maximizing lung cancer detection efficacy. It also recognizes the trade-offs between accuracy and efficiency when merging deep and traditional features, highlighting the importance of careful feature selection. Moreover, addressing the challenges in this integration and investigating hyper-parameter tuning for machine learning models may present avenues for future improvements.

## Introduction

Medical imaging analysis, including Computed Tomography(CT) is vital for detecting lung cancer. Medical diagnosis is primarily dependent on the careful examination of these medical images. However, there is still a significant gap in the field of medical diagnosis. Because of the images’ complexity and unique properties, analyzing and interpreting them is a specialized job that is typically reserved for experts. Based on statistics of The Global Cancer Observatory (GCO), lung cancer remains the primary cause of death, responsible for approximately 2.21 million fatalities, which accounts for 18% of all cancer-related deaths^1^. Each year, lung cancer claims the lives of over 7.6 million people globally. The cases of lung cancer increased, from 16,596 to 29,576 per year in Egypt^2^. Delayed detection of lung cancer causes an increase in death rate among lung cancer patients. So, programmers work hard to help in early detection of lung cancer to save lives^3^.

Artificial intelligence (AI) is considered an essential part of the field of computer science which aims to help computers think and solve the issues^4^. herefore, advancements in machine learning and deep learning with integrated AI have significantly impacted medical image processing and classification. In the field of medicine, this integration helps with the identification of abnormal nodules, while radiologists might find it challenging to diagnose or time-consuming to identify by utilizing of AI algorithm^5^. Furthermore, these technologies enable radiologists to make predictions, allowing for preventive measures to be taken before the disease manifests. And among these AI of algorithms that help radiologists in diagnosis are Reinforcement Learning (RL); Active learning integrated with deep learning and machine leaning.

In recent years, deep learning has significantly enhanced the effectiveness of computer-aided diagnosis (CAD) algorithms for cancer screening^6^. Nonetheless, a drawback is associated with numerous deep learning classification models, including convolutional neural network (CNNs) and Convolutional-Attention Network(CoAtNet). CNN i refers to convolutional neural network which begins with a matrix of input image and then extracts the critical features from the image layer by layer. CNN architecture consists of 3 basic parts: convolution layer, pooling layer, and fully connected layer. In the convolution layer the image is extracted by the convolutional filtering of the input image. Then, the pooling layer reduces the dimension of extracted features while preserving the main characteristic of the image. Finally, the fully connected classifies the medical image as cancer or non-cancer.^7^.Advanced models utilizing CNNs have been successfully applied to a variety of medical image analysis tasks, including disease detection from X-ray images, further demonstrating their versatility in the medical domain^8^).

CoAtNet Model refers to Convolutional-Attention Network. This is a hybrid model which combines between Convolutional Neural Networks and Transformer (ViT). CNN algorithms have detected important features of image like the edges, textures, and corners. Additionally, the transformer corner captures the data of regions of the picture which are a long way apart from each other by using Self-Attention Layers. CoAtNet has a number of stages. Firstly, the initial stages use convolutional layers and max pooling layers to extract the low features from images and map the reduced features. Secondly, the middle stages are combined with convolutional layers and self-attended to extract local features of the organ and distribution of lung tissues. The last stages refer to using the self-attention layers to extract more of the global features and classification of dataset^9^.

A critical challenge with these sophisticated deep learning and hybrid models is their dependency on vast, expert-annotated datasets to attain optimal accuracy. This necessitates extensive datasets meticulously annotated by expert radiologists, which is a persistently costly, time-consuming, and labor-intensive process, creating a significant labeling bottleneck, particularly in medical imaging analysis where enormous amounts of unlabeled data exist but individual annotation is impractical. Therefore, active learning is used by selectively choosing which data points from an unlabeled dataset should be labeled. It does this by iteratively selecting the most informative, uncertain sample of dataset. By focusing on these specific samples, active learning aims to supply the model with the information it requires to generalize more effectively while reducing labeling costs and time^10^. Active learning maximizes the use of some query strategies of active learning such as least confidence, entropy, and margin. Moreover, current systems often rely on either hand-crafted features or deep learning-derived features, but rarely optimize the synergistic potential of multiple, diverse feature extraction methods. A key challenge lies in effectively fusing, selecting, and leveraging these heterogeneous feature sets without introducing redundancy or noise, especially with the high dimensionality inherent in comprehensive medical image analysis. Many existing approaches either oversimplify feature representation or lack a systematic way to identify the most pertinent attributes across different feature spaces, leading to suboptimal classification performance and missed diagnostic cues crucial for early and accurate detection. In addition to active learning, reinforcement learning has improved the computer analysis of medical pictures in recent years. It is used to analyze medical images, solve problems related to image analysis, and classify images. Reinforcement learning also can help make the best decisions to classify the medical image. Deep Q-learning and deep Q-Network are some types of deep reinforcement learning^11^. However, designing an RL framework that can effectively learn optimal policies within the constraints of medical image analysis, without requiring prohibitive amounts of trial-and-error in a clinical setting, remains a complex challenge. A critical unmet need is for diagnostic systems that can adapt and improve continually based on new, diagnostically challenging cases, simulating a radiologist’s evolving expertise. Explainable AI (XAI) is used in deep learning (AI) and Machine Learning (ML) which helps the radiologists make decisions. Many researchers realized the significance of explanation, so the AI models of explainable lung nodules diagnostic is developed by predicting the Clinical features. The explainable AI can be used for detecting useful features and information in medical images. Wherefore it becomes most important in artificial intelligence^12^.The application of XAI is crucial for ensuring the interpretability of machine learning decisions in clinical practice, having been effectively demonstrated in diagnostic tasks such as stroke detection, where models like Random Forest are combined with SHAP XAI to highlight influential features^13^). ML techniques applied to medical images are crucial for efficient and cost-effective information extraction from these images. Such techniques greatly enhance the capacity of researchers and healthcare professionals to comprehend the underlying factors contributing to various illnesses. Some notable methods encompass eXtreme Gradient Boosting(XGBoost), random forests (RF), K-nearest neighbors (KNN), and decision trees (DT).

Despite the promising advancements in deep learning models, particularly hybrid architectures like CoAtNet, a significant hurdle to their widespread clinical adoption remains: the ‘black box’ nature. Radiologists often hesitate to fully trust AI recommendations without a clear, interpretable explanation of why a particular diagnosis was made. This trust deficit is exacerbated in multi-faceted hybrid models where feature extraction is distributed and complex, making it difficult to pinpoint the exact visual cues influencing the decision. The lack of transparent reasoning not only hinders clinical integration but also complicates error analysis and model refinement, creating a critical need for robust Explainable AI (XAI) tailored for these sophisticated architectures. The goal of AI in medical imaging is not just to classify, but to integrate seamlessly into clinical workflows and enhance diagnostic confidence. A significant gap exists in creating and validating holistic AI-driven diagnostic pipelines that not only achieve high technical accuracy but also provide actionable, interpretable insights directly validated by expert radiologists. Many research efforts focus on individual components (e.g., a new classification model or an XAI technique) but fail to integrate these into a coherent system where active learning efficiently curates’ data. Multiple feature types are optimally exploited, hybrid deep learning models perform classification, and XAI outputs are clinically vetted and refined based on radiologist feedback. This comprehensive integration, coupled with expert opinion, is essential to bridge the chasm between research prototypes and real-world clinical utility.

The objective of this research is to establish a comprehensive machine-learning workflow integrating active reinforcement learning, preprocessing, feature extraction, feature selection, and classification techniques with XAI for precise detection of lung cancer from CT scan images. The study concentrates on refining the efficiency and efficacy of the classification model through active learning, diminishing the necessity for extensive labeled data. Additionally, there is a specific emphasis on preprocessing to enhance image quality, involving resizing, noise removal, and employing various methods for feature extraction. Exploration of feature selection techniques aims to identify the most pertinent attributes, optimizing model performance for the classification task. Two primary classification approaches are investigated: traditional machine learning algorithms with diverse feature sets and deep learning models that integrated the CoAtNet architecture with explainable AI (XAI) by using large dataset CT image lung cancer. In Addition, the attention fusion is integrated with CNN and CoAtNet models to improve accuracy. Evaluation metrics encompass accuracy, training and testing times, and Area Under the Curve (AUC) scores, providing valuable insights into the most suitable techniques for accurate lung cancer detection. The overarching goal of this research is to make a substantive contribution to the field of medical image analysis by identifying the most effective combination of methods to enhance diagnostic accuracy in lung cancer detection. This research paper significantly advances the domain of lung cancer diagnosis using CT images by addressing several pivotal challenges. The key contributions in this paper include the following:

1. The paper focuses on leveraging a substantial dataset to improve the accuracy of lung cancer detection models. Utilizing a large dataset enables the model to learn from a diverse range of cases, fostering better generalization and ultimately achieving higher accuracy in both training and testing phases.
2. The paper also addresses the challenge of labeling data, traditionally a time consuming and expensive process. To address this issue, it implements active reinforcement learning, intelligently selecting informative samples for manual labeling. This approach significantly reduces the time and resources required to label a large, unlabeled dataset, thereby enhancing the efficiency of the training process.
3. To improve the result of deep learning, the research employs various algorithms for feature extraction and selection. Careful selection and optimization of features enhance the effectiveness of the classification process and also, combines the attention fusion feature with different architectures of deep learning.
4. Finally, CoAtNet is integrated with explainable AI to help experts diagnose and provide more useful information in CT images and take the decision of diagnosis with the Radiologist evaluated to enhance the result.

This study falls into the following categories: section “Related works” discusses the related works. Section “Dataset for lung cancer” collects the dataset used in this study. Section “Methodology” describes the process. Section “Experiments and results” describes the experiments and outcomes. Section “Conclusion” and “Future works” present the conclusion and future work of this paper.

## Related works

Many researchers have put their heads together to develop an effective and efficient approach for detecting or forecasting lung cancer as well as increasing the true-positive rate of lung nodules. These techniques include algorithms for image processing, deep learning detection, and machine learning. These procedures are used on CT images to determine the most efficient and accurate lung cancer detection outcomes.

Luo et al.^14^ proposed a new approach to the LLC-QE model which integrates reinforcement learning and ensemble learning for classifying lung cancer. The Artificial Bee Colony (ABC) algorithm is used to reduce the probability of the model getting stuck in the optimum of local. The feature vectors are extracted by using the CNN model. To train and assess this model, the Lung Image Database Consortium and Image Database Resource Initiative (LIDC-IDRI) dataset which contained predominately the set of cases without cancer, was used. Reinforcement learning formulated is used as a series of decisions interconnected to reduce the imbalance of the dataset. The F-measure of LLC-QE achieved 89.8% and a geometric mean of 92.7%.

Saha et al.^15^ introduced the new Volumetric Encoder-Decoder Residual Network(VER-Net) using three different transfer learning models to detect lung cancer in CT images. The dataset from Kaggle includes 1,653 CT images. The results show that the VER-Net model reached a high accuracy of 91% when compared to other models.

Bhatia et al.^16^ authors tackled the trade-off between computational cost and performance by introducing a Lightweight Advanced Deep Neural Network( DNN). Aimed at resource-limited environments, the model was trained on the LUNA16 dataset—a curated subset of LIDC-IDRI containing 888 CT scans—with a focus on low memory use and noise reduction. It achieved a high accuracy of 98.2%, showing that streamlined, single-stream architectures can perform on par with more complex ensemble models. That said, like Shatnawi et al.^17^ , the small dataset makes it difficult to fully evaluate the model’s robustness across diverse clinical scenarios. Additionally, while the approach is computationally efficient, it emphasizes performance metrics and does not include Explainable AI (XAI) visualizations to confirm the clinical relevance of the features it learned.

Shatnawi and Abuein^17^ developed models for automatic prediction and classification of lung cancer CT scan images. They used a dataset of 1,000 CT scans from Kaggle, which includes four types of cancer: 215 normal images, 187 large cell carcinomas, 338 adeno-carcinomas, and 260 squamous cell carcinomas. The data was split into 70% for training and 30% for testing, ensuring a balanced dataset. This research employed several pretrained models, including ConvNeXtSmall, InceptionV3, ResNet50, EfficientNetB0, and VGG16, with testing accuracies of 87%, 76.9%, 94.5%, and 97.9%, respectively. Additionally, the customized CNN model achieved a testing accuracy of 100%, outperforming the other models.

Ahmad Hassan et al.^18^ improved a Gestational Diabetes Mellitus (GDM) prediction model using a fusion technique that combines multiple algorithms with explainability. Given the significant risks linked to GDM, they proposed a new way to build a prediction model that merges traditional Machine Learning (ML) methods with cutting-edge Deep Learning (DL) algorithms. The hybrid model uses several ensemble methods and a meta-classifier to deliver reliable prediction performance. They applied data preprocessing techniques like multiple imputation, feature engineering, and oversampling to tackle class imbalance before running the model. These efforts resulted in high performance levels: accuracy at 98.21%, precision at 97.72%, and AUC at 99.91%, all of which surpass earlier studies using the same data. The authors use explainable AI (XAI) methods to highlight the most important features. This improves interpretability and supports proactive GDM management that can enhance maternal and fetal health.

The totality of the advanced methods in Table 1 shows a clear trade-off for clinical practice concerning annotation and computation costs as well as interpretability. Ensemble-based systems like VER-Net^15^ rely on large, fully annotated datasets and carry heavy inference costs because of multi-stream processing. On the other hand, custom and lightweight CNNs^16,17^ achieve impressive computational efficiency and high reported accuracy, but they often rely on very limited datasets, sometimes with fewer than 1,000 images. This raises concerns about generalizability and potential overfitting. More complex pipelines, such as LLC-QE^14^, add significant architectural complexity through reinforcement learning. This can make the models harder to audit. In the medical prediction field, models like the GDM prediction fusion model^18^ have shown high performance and included XAI for interpretability, but these methods usually focus on tabular datasets, which may be imbalanced instead of focusing on image-based diagnostics.

Importantly, none of these methods address the three main challenges in lung cancer CAD systems: (1) reducing reliance on large, annotated datasets, (2) maintaining computational efficiency for use in resource-limited settings, and (3) improving interpretability to build trust among radiologists. This gap motivates the proposed approach, which combines Active Learning with Reinforcement Learning and XAI-guided feature selection. This combination aims to lower labeling costs, improve diagnostic transparency, and achieve strong performance while keeping computational demands low.

**Table 1:** Comparison of State-of-the-Art (SOTA) methods highlighting data size, computational cost, and interpretability.

Refer.& method	Year	Annotated data	Performance	Comput. cost	Interpretability & limitations
Luo et al.^14^ (LLC-QE)	2023	LIDC-IDRI imbalanced.	F-Measure: 89.8% Geo-Mean: 92.7%	**Very High:** Complex integration of Reinforcement Learning and ABC optimization	**Complex:** Dynamic decision paths via RL make the model opaque and hard to audit
Saha et al.^15^ (VER-Net)	2024	1,653CT Images (Kaggle)	Accuracy: 91%	**High:** Requires inference of 3 RL models (ensemble)	**Low:** Ensemble voting obscures specific visual features; no explanation provided
Bhatia et al.^16^ (Lightweight DNN)	2024	888-CT Images. (LUNA16 )	Accuracy: 98.2%	**Low:** Designed specifically for low memory and computational efficiency	**Low:** Solves the cost issue but lacks XAI visualizers (Grad-CAM) for transparency
Shatnawi et al.^17^ (Enhan. CNN)	2025	1,000CT Images. 4 Classes	**Accuracy:** 100%Enhan. CNN 99% VGG16 97.9%EfficientNetB0	**Low/Moderate:** Authors claim “rapid analysis,” though enhancement layers add preprocessing	**Very Low:** 100% accuracy on small data suggests *overfitting*. No XAI mechanism for clinical transparency
A. Hassan et al.^18^ (Fusion Model)	2025	GDM Imbalanced.	Accuracy: 98.21% AUC: 99.91% F1-score: 97.59%	**Moderate:** Fusion of ML and DL algorithms; oversampling	**High:** Utilizes XAI techniques to interpret model decisions and highlight influential features

## Dataset for lung cancer

**Fig. 1:**
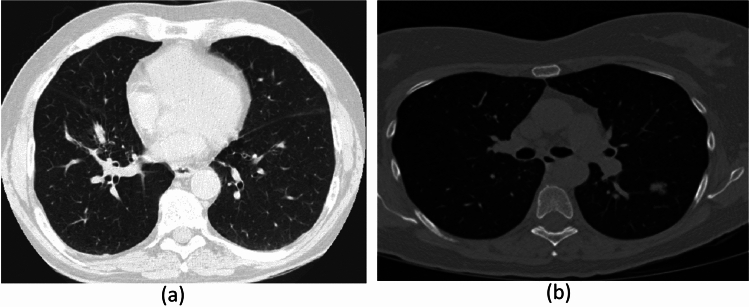
sample of dataset CT image Kaggle Bowl2017, (**a**) normal, (**b**) abnormal.

This research uses a curated subset of the Data Science Bowl 2017 dataset^19^. From the original 285380 CT images for 2101 patients, 30,020 CT images belonging to 790 patients is selected. This dataset was divided into labeled and unlabeled sets to assess model performance^20^. Figure 1 provides sample images.

## Methodology

**Fig. 2:**
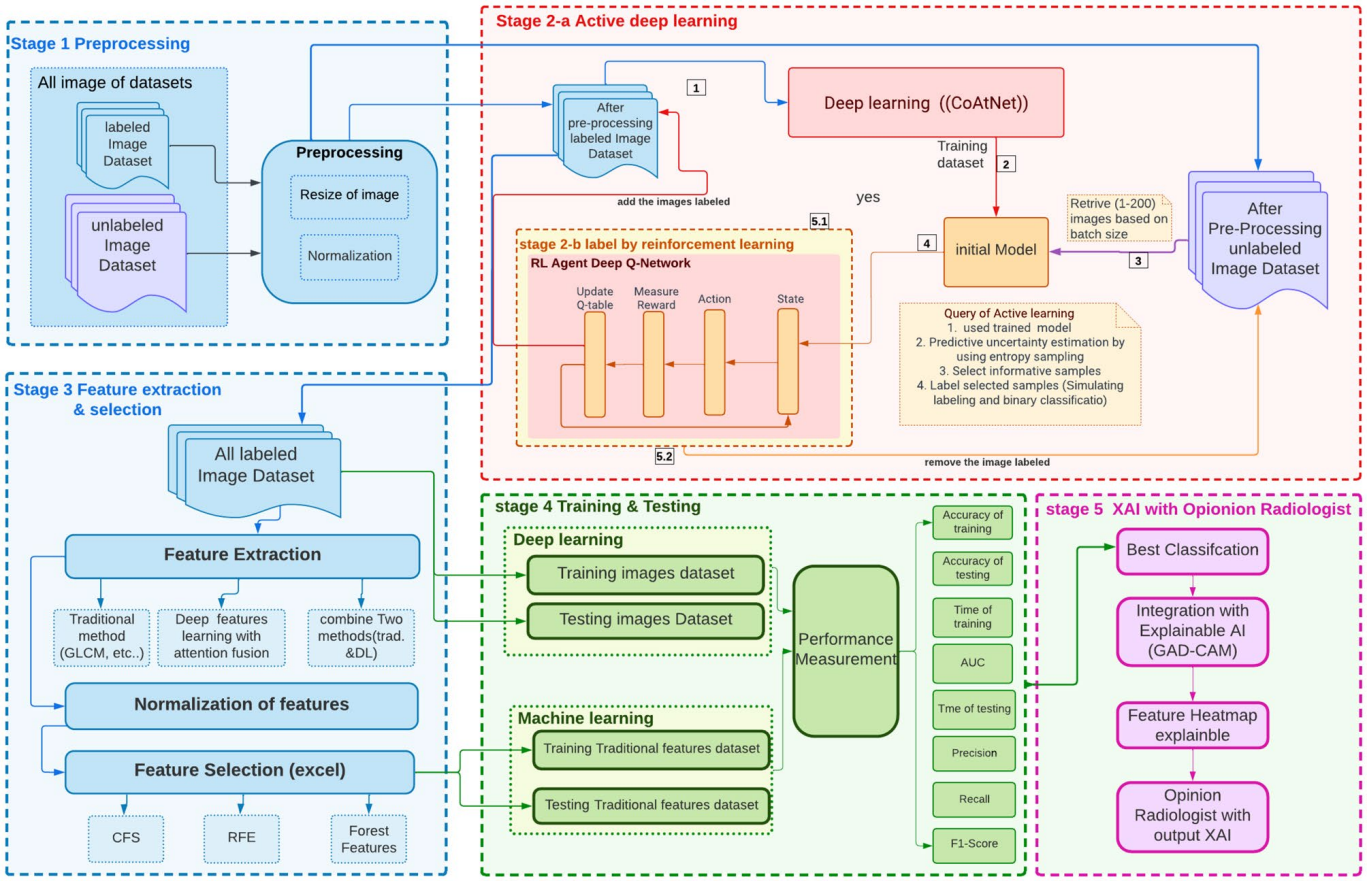
Block diagram of the proposed model.

The provided block diagram outlines the proposed model: Active reinforcement learning and explainable AI with deep attention to Fusion Features (ARXAF-Net) where it is a multi-stage process for active reinforcement deep learning with XAI, as shown in Fig. 2.

In Stage 1, the preprocessing phase commences with two distinct datasets: a labeled image dataset and an unlabeled dataset. Stage 2-(a, b) presents an active reinforcement deep-learning process following preprocessing. Stage 3 focuses on feature extraction by traditional techniques and deep feature learning model. In Stage 4, the process concludes with training and testing, which includes evaluating performance metrics for both training and testing datasets using traditional machine learning methods and deep learning models. Stage 5 presents the integration between best model of stage 4 with explainable AI to help experts with the decision making of diagnosis CT image and then give it to radiologist to evaluate the result of XAI by uisng Gradient-weighted Class Activation Mapping (GRAD-CAM).

### ARXAF-Net algorithm overview

The ARXAF-Net model comprises five core stages that integrate preprocessing, active deep learning with reinforcement learning (RL), explainability (XAI), and robust performance evaluation as shown Algorithm 1.

**Algorithm 1:**
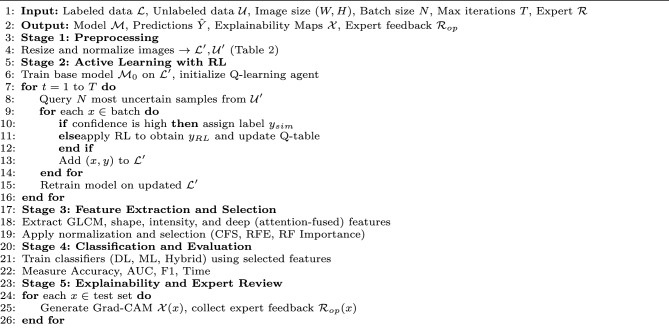
ARXAF-Net: Active Deep Learning with RL and XAI.

#### Stage 1-preprocessing of the dataset

In this initial stage, the total dataset contains 30,020 CT images, evenly balanced with 15,010 cancer images and 15,010 non-cancer images. Preprocessing commences with two distinct and essential datasets: a labeled image dataset and an unlabeled dataset, the characteristics of which are summarized in Table 2. The labeled dataset consists of 6,080 images, meticulously acquired from a total of 160 patients, precisely balanced with 80 cancer patients and 80 non-cancer patients. Each patient contributed 38 images, thereby ensuring equitable representation across the cohort. Specifically, 3,040 of these images are unequivocally classified as cancerous, while the remaining 3,040 are designated as non-cancerous, clearly demonstrating a balanced class distribution. To rigorously prevent patient level data leakage, the dataset was strategically split at the patient level into distinct training, validation, and test sets. This critical step ensures that no images from a single patient appear in more than one set. Furthermore, these splits were carefully stratified to maintain consistent class balance across all partitioned sets. As an integral part of the preprocessing pipeline, all images undergo a resizing operation to achieve a uniform resolution of 128 $\times$ 128 pixels. Following this, the pixel intensity values are normalized across the entirety of the dataset to ensure consistency and minimize inter-image variation.

**Table 2:** Labeled and unlabeled CT image counts.

Dataset	Number of images
Labeled data	6080
Unlabeled data	23,940

#### Stage 2 - active reinforcement deep learning

Following initial preprocessing, a CoAtNet-based deep learning model is trained on the labeled dataset, resulting in a baseline classifier. The unlabeled dataset is then processed in batches of 200 images. For each batch, the model predicts class probabilities, and the entropy of each sample is computed to quantify prediction uncertainty:

$$\begin{aligned} H(x) = -\sum _{c} p_c \log (p_c) \end{aligned}$$

where $p_c$ is the predicted probability for class $c$. The top-k most uncertain samples, determined using a predefined entropy threshold, are selected and passed to a reinforcement learning (RL) agent for pseudo-labeling.The pseudo-labeling process is formalized as a Markov Decision Process (MDP):

$$\begin{aligned} \text {MDP} = (S, A, P, R, \gamma ) \end{aligned}$$

where:

- **State** $S$: the flattened feature vector of the selected image.
- **Action** $A$: label assignment (0 = non-cancer, 1 = cancer).
- **Transition** $P(s'|s,a)$: determined by moving to the next image in the batch.
- **Reward** $R(s,a)$: $+1$ if the assigned label matches the predicted label, $-1$ otherwise.
- **Discount factor** $\gamma$: 0.95, controlling the importance of future rewards in Q-value updates.

In this framework, the RL agent performs label selection, not sample selection, which distinguishes it from standard uncertainty sampling. The transition $P(s'|s,a)$ follows the sequential order of the top-k uncertain samples: after labeling sample $i$, the agent moves to the state of sample $i+1$. The RL agent learns a policy that maximizes label correctness and reduces noise in the pseudo-labeled dataset, something classical active learning cannot achieve. The Q-learning agent updates the Q-table iteratively using:

$$\begin{aligned} Q(s,a) \leftarrow Q(s,a) + \alpha \big [ r + \gamma \max _{a'} Q(s',a') - Q(s,a) \big ] \end{aligned}$$

where $\alpha = 0.1$ is the learning rate, $r$ is the reward, $s'$ is the next state, and $a'$ is the next action. The reward function is formally defined as:

$$\begin{aligned} R(s,a) = {\left\{ \begin{array}{ll} +1, & \text {if the pseudo-label matches the CoAtNet model's predicted class}\\ -1, & \text {otherwise} \end{array}\right. } \end{aligned}$$

This reward structure guides the agent to generate pseudo-labels consistent with high-confidence model predictions. Unlike rewards tied to global metrics, this **per-sample feedback** ensures the agent prioritizes data integrity. The goal is to train an RL agent that:

1. Reduces noise in the pseudo-labeled dataset,
2. Maintains consistency with model confidence, and
3. Selects the most reliable and informative samples.

By encouraging high-quality pseudo-labeling, the reward function indirectly enhances the CoAtNet classifier’s accuracy and stability as it is retrained on a progressively cleaner dataset.

After pseudo-labeling, the new samples are incorporated into the labeled pool:

$$\begin{aligned} X_{\text {labeled}} \leftarrow X_{\text {labeled}} \cup X_{\text {pseudo}}, \quad y_{\text {labeled}} \leftarrow y_{\text {labeled}} \cup y_{\text {pseudo}} \end{aligned}$$

At the end of each iteration, the CoAtNet model is retrained on the expanded dataset. This loop continues for up to 120 iterations, ensuring convergence of both Q-values in the RL component and the overall model performance. Unlike standard active learning, which only selects uncertain samples, this framework allows the RL agent to learn an optimal labeling policy, reducing pseudo-label noise and improving the quality of the expanded training set.

In summary, Stage 2 integrates uncertainty-based active learning with reinforcement learning under a formally defined MDP. The framework is characterized by well-defined states, actions, rewards, and Q-learning updates. Hyperparameters are explicitly set, including a batch size of 200, Q-learning rate $\alpha = 0.1$, discount factor $\gamma = 0.95$, and exploration rate $\epsilon = 0.1$. Through strategic interaction, the agent effectively identifies the most informative samples, leading to significant improvements in model accuracy and providing a clear advantage over conventional active learning, reinforcement learning alone, or random sampling approaches.

#### Stage 3-a feature extraction

Feature extraction from CT lung cancer images is a critical process. The feature extraction process is for a dataset of 30,020 images. Below, we’ll discuss each of these feature extraction categories:

- **Texture features:**^21^ is GLCM and LBP. Local Binary Patterns are referred to as LBP which yield 25 distinct features. GLCM refers to Gray-Level Co-occurrence Matrix. GLCM contributes a further 15 features, including contrast, dissimilarity, homogeneity, energy, and correlation, each calculated for three different directions
- **Shape features**^22^**:** provide geometric characteristics of objects in CT images, including area, perimeter, and compactness.
- **Intensity-based features**^22^**: ** The five features are calculated, including intensity of mean, standard deviation, median, skewness, and kurtosis.
- **Deep features with attention fusion features:** channel attention is first computed by applying global average pooling across the feature maps, followed by a two-layer Multi-Layer Perceptron(MLP ) and a sigmoid activation to generate channel-wise weights. Spatial attention is then calculated by concatenating the average-pooled and max-pooled features along the channel dimension, passing them through a 7$\times$7 convolution, and applying a sigmoid activation. The resulting attention map is applied multiplicatively to the feature maps. Channel and spatial attention are applied sequentially, following the standard procedure described in the referenced method.^23^.
- **Combining feature (Traditional + Deep):** All feature vectors—Texture, Shape, Intensity, and CNN—are standardized to have zero mean and unit variance to ensure they are on comparable scales. The CNN features are first passed through an attention weighting mechanism, where each feature dimension is multiplied by a learned weight normalized via a sigmoid function. This approach emphasizes the most useful deep features and reduces the impact of irrelevant ones. Since the attention weights range from 0 to 1, no CNN feature can exceed the dynamic range of the standardized handcrafted features. This stops any single feature from dominating and keeps the fusion balanced. After applying the attention weights, we combine the CNN features with the standardized traditional features to create a single 176-dimensional feature vector for each image: 40 (Texture) + 3 (Shape) + 5 (Intensity) + 128 (CNN) = 176 features. This combination reflects an early fusion method, where we merge all feature types before classification instead of combining them at the decision level.
  This method guarantees a balanced representation. The attention mechanism highlights important CNN features without allowing them to overpower others, while standardization ensures that all feature types have a similar role in the classification process.

#### Stage 3-b normalization of feature extraction

Normalization is a crucial preprocessing step when dealing with diverse types of features in machine learning to consistent scale of features, including texture, shape features and intensity-based features.

#### Stage 3-c features selection

This paper focuses on the use of feature selection strategies to increase the performance of machine learning. Three different feature selection approaches were used to sort and prioritize a dataset’s most pertinent attributes, which assign the score to each utilized attribute. Higher relevance ratings are retained, whereas lower significance parts are eliminated. IThe features evaluated for their effectiveness are forest feature (FF), correlation-based feature selection (CFS), and recursive feature elimination (RFE).

#### Stage 4-a classification

To ensure a rigorous and unbiased evaluation, all dataset splits were performed strictly at the patient level rather than the image level. The dataset consists of 790 patients, each contributing approximately 38 CT images, for a total of 30,020 images. Two patient-level splits were performed: the first for ARL evaluation using only the initially labeled subset (160 patients), and the second for final ML/DL classification after all images were labeled (all 790 patients). For the Active Reinforcement learning (ARL) framework, 160 patients (6040 images) were assigned to the labeled set, and unlabeled pool is 630 patients( 23940 images). Subsequently, the labeled set was divided into training (70%—112 patients, 4256 images), validation (15%—24 patients, 912 images), and testing (15%—24 patients, 912 images). Furthermore, to make sure that the test set images were never used in labeling, training, or validation, a final held-out test set was carefully separated from all ARL iterations and the initial model training. This prevents any data leakage and guarantees that the model is evaluated on completely unseen patients, providing a realistic measure of its generalization performance. Three approaches were implemented as follows:

The first approach, the classification of machine learning models, comes into play after extracting features from the CT input to discern between cancerous and normal cases. It employs various classification techniques, including XGBoost, RF, model with used attention fusion features, second approach and high accuracy of first Bayesian network, and DT methods.

The second approach, various deep learning architectures for feature extraction from the input images are assessed, such as a CNN with attention fusion, CoAtNet, and a Simple CNN.

- **Simple CNN:** includes a global average pooling layer that generates a 128-dimensional feature vector, as well as three convolutional layers (32, 64, and 128 filters of size 3$\times$3), each followed by max pooling. There are 92,672 total trainable parameters.
- **CoAtNet:** Identifies local and global features by combining convolutional and attention mechanisms. In order to generate a 128-dimensional feature vector, the network employs three convolutional layers with 128 filters each (3$\times$3), interspersed with max pooling and dropout layers, and then global average pooling. 296,448 are the total trainable parameters.
- **Simple CNN with attention fusion:** This improves the Simple CNN by adding a mechanism for attention fusion. The input image’s features are extracted by two CNN branches operating in parallel. Channel attention, which highlights the most informative feature channels, and spatial attention, which highlights significant spatial regions, are then used to combine these features. The final output is produced by pooling the fused feature maps using global average pooling and passing them through fully connected layers (Dense 128 $\rightarrow$ Dropout $\rightarrow$ Dense 64 $\rightarrow$ Dense 1).

Table 3 summarizes the specific hyperparameters for each model, including the number of layers, filter sizes, activation functions, dropout rates, optimizer, number of epochs, classifier type, loss function, and attention mechanism. This ensures reproducibility and helps clarify the technical implementation of the attention fusion mechanism. To identify the most suitable architecture for the study’s overall strategy, all models were trained under the same conditions and their performances were compared.

The last approach, the combination between the high results of deep learning approach of traditional features. To gauge the performance of these techniques by metrics in section “Stage 4-b performance metrics”.

**Table 3:** Hyper-parameters of the deep learning architectures.

Hyperparameter	Simple CNN	CoAtNet (Simplified)	CNN with attention fusion
No. of layers	3 Conv+3 Pool+3 Dense	3 Conv+2 Dropout+3 Dense	2 Conv branches+2 Attention+3 Dense
Filter sizes	3$\times$3	3$\times$3	3$\times$3
Activation function	ReLU	ReLU	ReLU
Dropout rate	0.5	0.5	0.5
Optimizer	Adam	Adam	Adam
No. of epochs	60	60	60
Classifier	Sigmoid	Sigmoid	Sigmoid
Loss function	Binary Crossentropy	Binary Crossentropy	Binary Crossentropy
Attention mechanism	None	Built-in attention fusion	Channel + Spatial Attention Fusion

#### Stage 4-b performance metrics

Different performance measures are being used to assess the effectiveness of machine and deep learning models derived from true positives (TP), true negatives (TN), false positives (FP), and false negatives (FN)

- **Accuracy (ACC):** Calculate how accurate the model’s predictions are overall.
  1 $$\begin{aligned} ACC = \frac{TP + TN}{TP + TN + FP + FN} \end{aligned}$$
- **Precision** Precision measures the proportion of true positive predictions among all positive predictions.
  2 $$\begin{aligned} Precision = \frac{TP}{TP + FP} \end{aligned}$$
- **Recall** Recall measures the proportion of true positive predictions among all actual positives.
  3 $$\begin{aligned} Recall = \frac{TP}{TP + FN} \end{aligned}$$
- **F1-Score:** The F1-score which provides a balance between precision and recall.
  4 $$\begin{aligned} F1Score = 2 \cdot \frac{Precision \cdot Recall}{Precision + Recall} \end{aligned}$$
- **AUC-ROC** That indicates model performance where higher values indicate better performance.

#### Stage 5 explainable AI with deep learning from a radiologist viewpoint

In this study, the model was evaluated by three board-certified radiologists using a total of 912 CT images. A custom Python tool displayed each scan alongside its Grad-CAM heatmap, allowing the radiologists to review both the raw image and the model’s highlighted regions. Their feedback concentrated on three key areas:

- whether the marked regions corresponded to clinically significant tumor areas.
- whether any concerning regions were missed
- whether the model highlighted irrelevant features.

To evaluate the effect of explainable AI (XAI) on diagnostic accuracy, each radiologist assessed the scans two times—initially without Grad-CAM and then with the heatmaps displayed. The incorporation of Grad-CAM enhanced radiologist accuracy from 96.7% to 99.9% and decreased reading time by approximately 25%, demonstrating notable improvements in both efficiency and confidence.

Grad-CAM was also evaluated quantitatively. Across the same 912 CT images, the model’s attention maps achieved a mean IoU of 0.72 ± 0.08 against radiologist-annotated lesion masks, and the Pointing Game accuracy reached 0.91. Only a small number of cases (about 3–5, or 0.3–0.5%) involved lesions that were missed or not sufficiently highlighted, typically very small $(<3 \text { mm})$ or low-contrast nodules near the lung periphery. Inter-rater validation showed strong consistency among the radiologists, with Cohen’s kappa ranging from 0.88 to 0.91 for lesion detection and an ICC of 0.89 for lesion-size estimation.

**Fig. 3:**
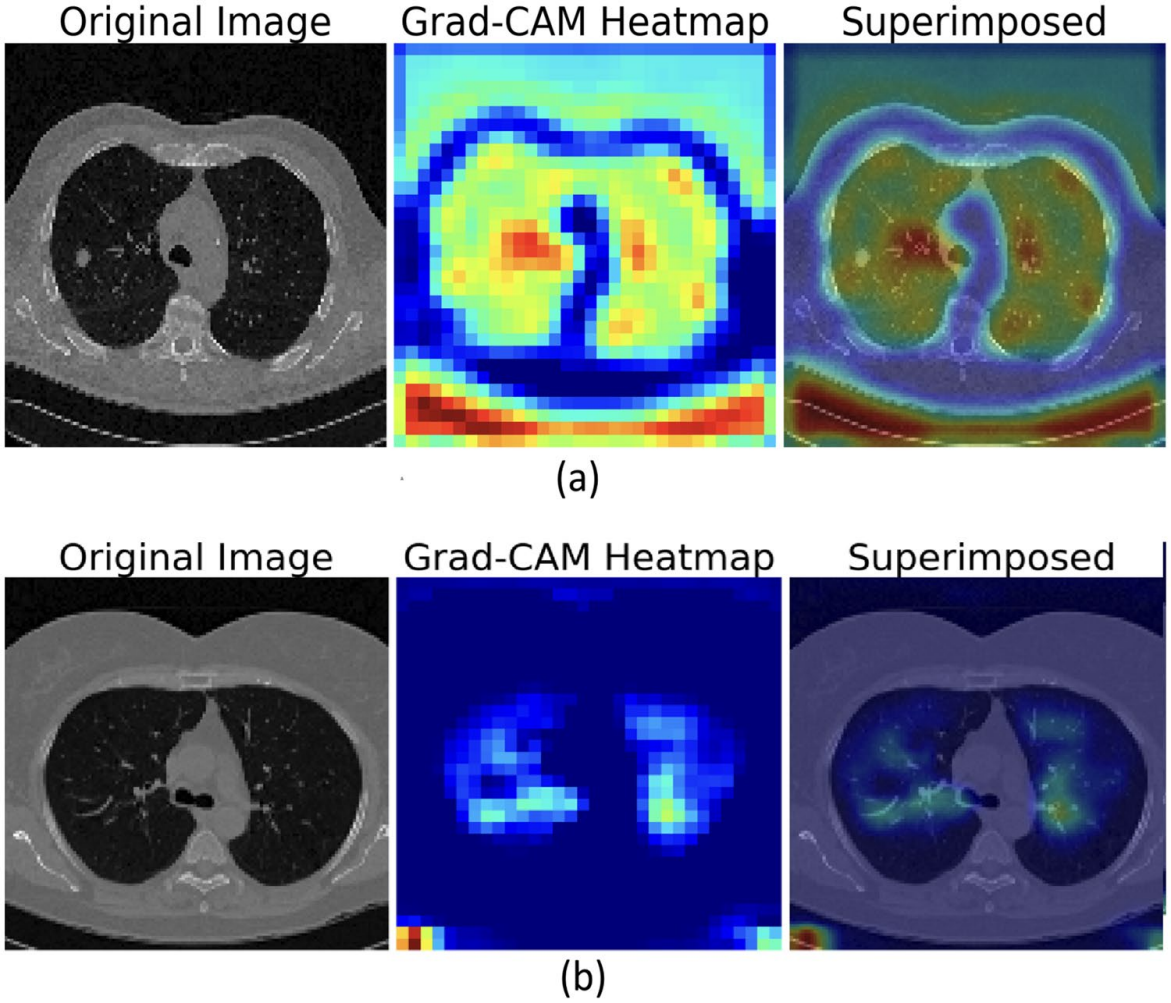
Sample of CT lung Image With XAI (Grad-CAM), (**a**) CT lung image Abnormal, (**b**) CT lung image Normal.

Qualitative analysis of the Grad-CAM maps shown in Fig. 3 that highlighted regions often corresponded to imaging features radiologists commonly use to identify malignancy, such as spiculation, irregular borders, or heterogeneous internal texture. Radiologist review confirmed that the model focused on the same diagnostic cues typically assessed in clinical practice, rather than irrelevant artifacts. Overall, the multi-radiologist XAI evaluation shows that the model attends to clinically meaningful regions, enhances radiologist performance, and maintains high interpretability, supporting its practical applicability in real clinical settings.

## Experiments and results

The computational setup for this experiment is as the following specifications:

- **Hardware:**
  - Using Lightning AI cloud Platform.
  - Nvidia L40S 48G (VRAM)
  - 16 GB GDDR6 9001 MHz
  - Operating System: Clould services
- **Framework:**
  - TensorFlow was utilized for the deep learning endeavors.

The experiment is divided into parts: the first part is about active reinforcement deep learning, and the second part uses the extracted traditional features for classification of all labeled datasets by different machine learning algorithms and combination with various deep learning models.

The first part discusses the extensive process of utilizing CoAtNet Architecture of deep learning and ARL techniques to enhance the performance of a medical image classification model. Firstly, the model begins by loading labeled and unlabeled medical images. Labeled data, presumed to have corresponding class labels (such as cancer and non-cancer), is resized to 128 x 128 pixels, and pixel values are normalized. Secondly, an initial CoAtNet model is constructed and trained on the labeled dataset. This model goes through 20 epochs, with a batch size of 64. The initial labeled data was divided into training 70%, validation 15%, and testing 15% subsets. The test set, containing 912 images from 24 patients, was strictly held out at the patient level and was never used at any point in the pipeline—including initial model training, active learning iterations, pseudo-labeling, Q-learning updates, or retraining of the model.

The initial model’s training accuracy, validation accuracy, and AUC score are all recorded. The initial training accuracy was roughly 68%, whereas the validating and testing accuracy were at 67.9%, 67.2% respectively as shown in Table 4 . Furthermore, the original of initial model’s AUC value of 75.1% demonstrates its ability to differentiate between positive and negative situations. Moreover, the precision score of 68.9%, the recall score of 69.8% , F1-score of 69.3%.

The initial model’s training accuracy, validation accuracy, and AUC score are all recorded. The initial training accuracy was roughly 68%, whereas the validating and testing accuracy were at 67.9%, 67.2% respectively as shown in Table 4. Furthermore, the original of initial model’s AUC value of 75.1% demonstrates its ability to differentiate between positive and negative situations. Moreover, the precision score of 68.9%, the recall score of 69.8%, F1-score of 69.3%.

Active Learning (AL) boosts performance by selecting the most informative, uncertain samples, which helps reduce model uncertainty and enhances generalization. However, since AL cannot correct noisy pseudo-labels, its improvement is naturally limited. Even so, it provides a realistic gain, with test accuracy increasing from 67% to 89.1% and AUC rising from 75.1% to 93.5%.

In the performance measurements of the final active reinforcement CoAtNet deep learning model. The model achieves a recall of 98.4%, the precision is high at 99.9% and the F-score is 98.4%. The model’s training accuracy is quite high at 99.8%, indicating its ability to fit the training data. In testing, the model exhibits strong performance with a test accuracy of 99.5%. The AUC is 99.8% which indicates its effectiveness in distinguishing between positive and negative cases.

**Table 4:** Performance metrics of the initial & final active reinforcement CoAtNet model.

	Recall	Precision	F1-score	Train Acc.	Test Acc.	AUC
Initial model	69.8%	68.9%	69.3%	68.0%	67.0%	75.1%
AL_Only(Final Model)	84.2%	86.7%	85.4%	88.9%	87.3%	92.4%
AL+RL(Final Model)	98.4%	99.9%	98.4%	99.8%	99.5%	99.8%

**Fig. 4:**
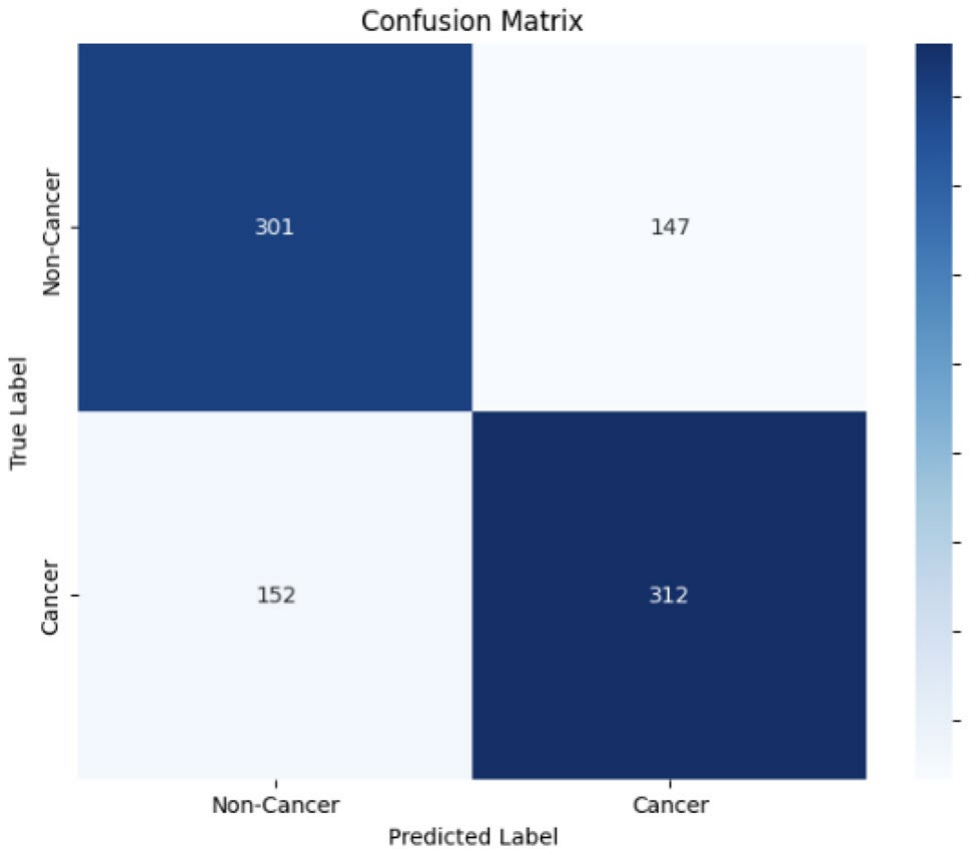
Confusion matrix of the initial of active reinforcement CoAtNet deep learning.

**Fig. 5:**
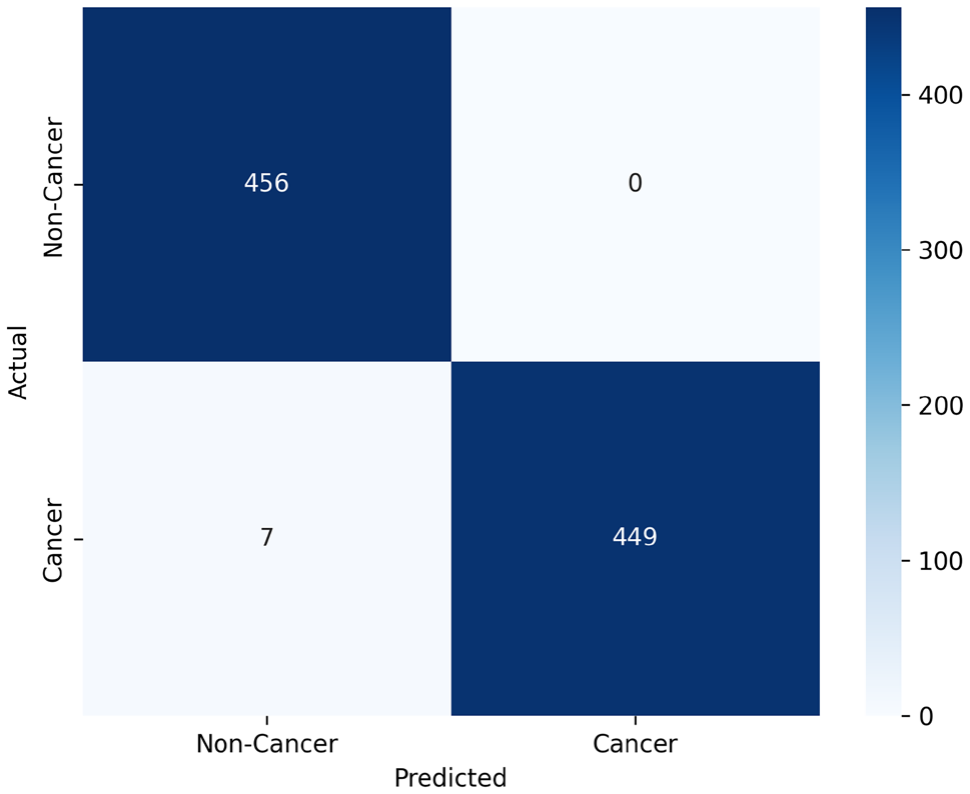
Confusion matrix of the final active reinforcement CoAtNet deep learning.

Figure 4 shows the initial model of confusion matrix in which the total number of testing data cases is 912. The confusion matrix in Fig. 5 for the final active reinforcement deep learning model presents a comprehensive view of its classification performance. The model’s predictions are evaluated against actual data. The total number of testing data cases is 912. The values in the matrix reveal that the model accurately identified 456 true negative cases. Additionally, it correctly identified 449 true positive cases. However, it made 7 false negative predictions, and non false positive predictions.

Table 5 shows the result of performance measurement of time of the initial & final model in the Active Reinforcement CoAtNet deep learning which contains 6080 labeled CT image. The results show that the model’s training, validating and testing time is 60.59, 0.259 s, and 0.26 s, respectively. While the training time of the final model is 23400 s, the testing time of all iterations is substantially lower at 142.34 s.

**Table 5:** Time performance measurement of Initial & Final ARL with CoAtNet Model.

Model	Train. time	valid. time	Test. time
Initial Model	60.59	0.259	0.262
AL_Only(Final Model)	11123.3	119.334	142.34
AL+RL(Final Model)	12676.98	119.334	142.34

**Fig. 6:**
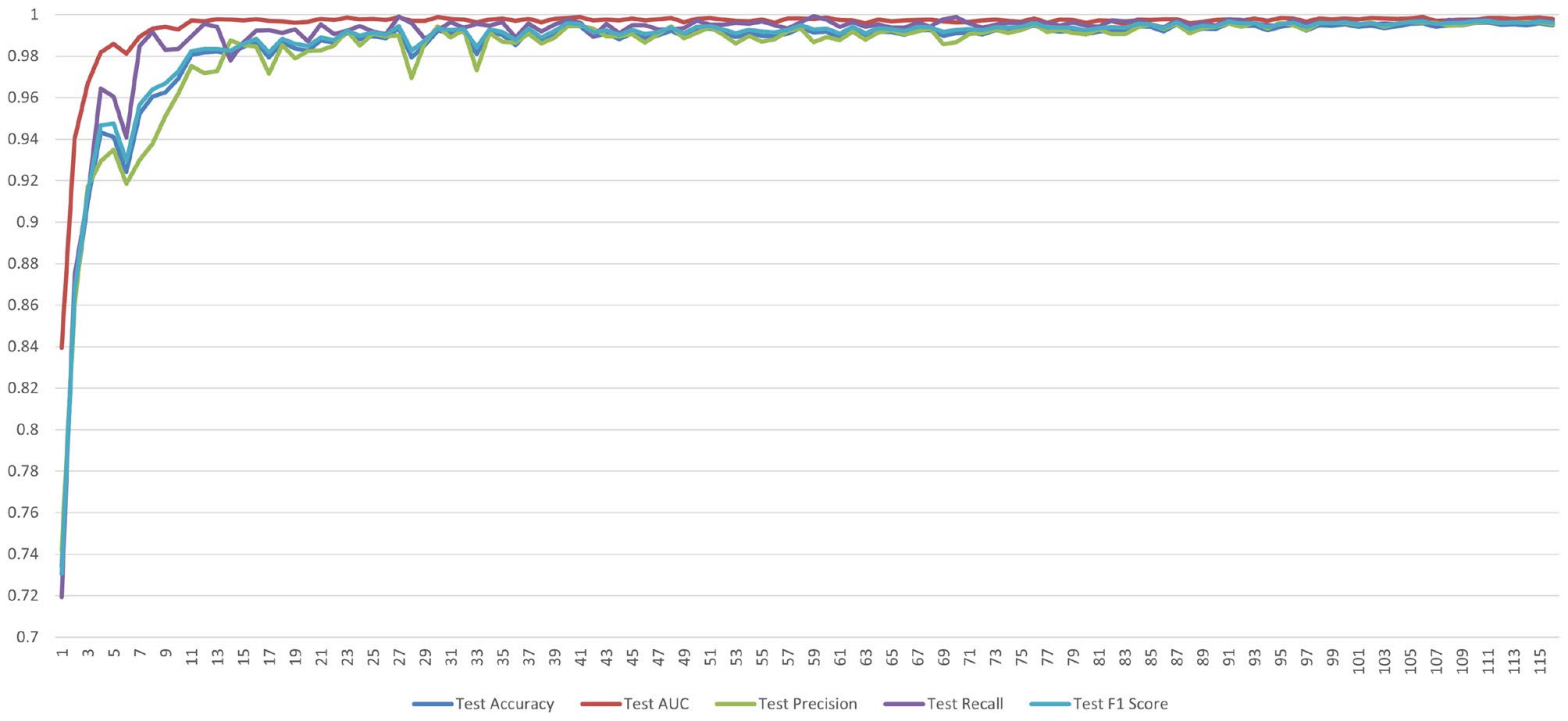
Performance measurement number of iterations in active Reinforcement CoAtNet learning.

Figure 6 presents the results of performance measurements for each iteration of ARL. It appears that the ARL process aims to improve the model’s performance over iterations by selectively choosing data points for labeling and training. In the initial query (iteration 1), the model demonstrates outstanding performance, achieving the value of accuracy of 73.0%, a precision of 74.1%, a recall of 71.9%, F1-score of 73.9%, and an impressive ROC AUC of 83.9%. After that, in each iteration of active reinforcement learning, it selects a batch of unlabeled data based on the model’s uncertainty (entropy of predictions) and then labels this batch. The goal is to prioritize labeling data that is most likely to improve the model. The labeled data from each iteration is added to the labeled dataset, and the model is retrained on the expanded dataset. Iterations 2, 3, 5, 6, and several others show consistent near-perfect results across all metrics, indicating the model’s ability to maintain a high level of performance as more labeled data is incorporated. Notably, in several iterations, the recall and ROC AUC values remain consistently high, which is indicative of the model’s ability to identify positive instances effectively. Then, various performance metrics are calculated after each iteration. The model maintains high overall performance, but with slight deviations in individual metrics.

In summary of the first experiment, the Active Reinforcement Deep Learning model exhibits significant improvement throughout the iterations. Active Learning combined with Reinforcement Learning (AL + RL) addresses AL’s main limitation by learning an optimal labeling policy that corrects pseudo-label errors and stabilizes uncertainty sampling. This ability to reduce noise explains the larger performance gain from AL to AL+RL, achieving 99.5% test accuracy and 99.8% AUC, making the final improvement both substantial and well justified. Also, as it shows lower testing time making, it proves to be a valuable tool for real-world applications.

After completion of the initial stage, where all CT images were meticulously labeled using the Active Reinforcement Learning (ARL) framework, all labeled dataset (30020 CT image) progressed to its second stage. This subsequent stage focused on extracting both traditional and deep features to facilitate the rigorous assessment of various machine learning and deep learning models in medical image classification. To ensure the integrity and prevent data leakage, the dataset was strategically partitioned at the patient level into 70% training (21,014 images,553 patients), 15% validation (4503 images,119 patients), and 15% testing (4503 images,118 patients) sets for all models. A crucial aspect of this stage involved a final, strictly held-out test set that remained entirely separate and was not utilized at any point during model development, thereby guaranteeing an unbiased evaluation of the models’ generalization capabilities. This comprehensive stage was executed in three approach:

- Firslty, **Traditional features and classification** are retrieved from pre-processed images. Texture features, shape features, intensity-based features, and correlation features are extracted from CT lung images. Next, Use the retrieved conventional characteristics to train and evaluate several classification algorithms such as XGBoost, Random Forest (RF), Decision Tree (DT), and Bayesian Network.
- **Deep feature extraction and Classification** uses a simple CNN model with attention fusion features, Simple CoAtNet and Complex CoAtNet with attention fusion feature models which developed for image categorization. Create an appropriate architecture using convolutional layers, pooling layers, fully linked layers, and an output layer. Train the deep learning model using the same data. Key hyperparameters such as the learning rate, optimizer, number of epochs, number of layers, and activation functions were meticulously fixed as shown Table 3. Evaluate the CNN model’s performance on the testing set using performance metrics.
- Finally, **Combined traditional features with deep features and classification** Based on a comparative analysis of accuracy and computational efficiency, the simple CNN with attention fusion was identified as the optimal deep learning model for integration with the previously extracted traditional features. The resultant fused model was then subjected to thorough evaluation on the testing set to ascertain the synergistic contribution of combining traditional and deep features. To understand how different feature types and architectural choices affect results, we conducted systematic ablations. We compared models using only traditional features, only deep features, and combined fused features. This analysis highlighted how feature selection methods, attention fusion mechanisms, and the complexity of the network architecture influenced the final classification performance.

**Fig. 7:**
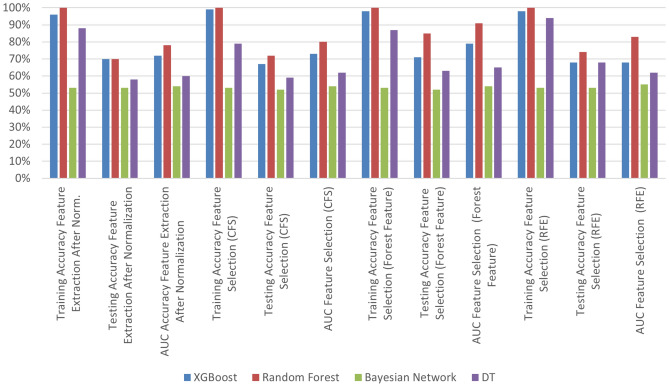
The accuracy training and testing and AUC of different classifiers.

First approach, Fig. 7 presents an extensive evaluation of machine learning algorithms for CT lung image for classification by various feature extraction and selection techniques. In the initial combination of 4 types of feature extraction phase after normalization, the XGBoost model achieved 96% training accuracy and 65% testing accuracy, while the Random Forest model demonstrated remarkable performance with 100% training accuracy and 70% testing accuracy. The Bayesian Network and DT models had relatively lower testing accuracy scores. In Forest Features extraction, the XGBoost model showed an improvement, achieving 96% training accuracy and 71% testing accuracy. The Random Forest model maintained its high accuracy, reaching 100% in training and 85% in testing.

After that, applying feature selection methods further refined the models. Using CFS method and FF method, the XGBoost model maintained 99% accuracy training and 67% testing, and the RF model achieved 85% testing accuracy while maintaining its perfect training accuracy. While RFE method, both XGBoost, DT and RF models achieved training accuracy of around 98%, 94% and 100%, respectively. Also, XGBoost, DT and Random Forest models of testing accuracies of around 68%, 68% and 74%, respectively, as shown in Fig. 7

Overall, the Random Forest model consistently outperformed other models in terms of accuracy and achieved impressive results, even after feature selection in CT lung image classification as shown in Table 6.

The consistently extremely low p-values across all metrics—Accuracy, AUC, Precision, Recall, and F1-score—in Table 7 provide strong statistical evidence that RandomForest_FF outperforms all other compared classifiers. Many p-values are effectively zero (for example, $1.52524\times 10^{-22}$ for Accuracy versus XGBoost_All, and 0.0 for Recall versus Bayes_All and DT_All), which clearly indicates that these performance differences are highly significant and not due to chance. This firmly supports the conclusion that RandomForest_FF is the superior model.

**Table 6:** Performance measurement with different ML and various of feature extraction & selection.

Features	Classifier	Train Acc.	Train time	Test Acc.	Test time	AUC
All features extract. After Normaliz.	XGBoost	96%	1.32 s	65%	0.01 s	72%
	Random Forest	100%	81.97 s	70%	0.31 s	70%
	Bayesian network	53%	0.04 s	53%	0.01 s	54%
	DT	88%	2.37 s	58%	0.01 s	60%
Feature selection (CFS)	XGBoost	99%	1.19 s	67%	0.01 s	73%
	Random Forest	100%	46.39 s	72%	0.24 s	80%
	Bayesian network	53%	0.03 s	52%	0.01 s	54%
	DT	79%	2.42 s	59%	0.01 s	62%
Feature selection(Forest Features)	XGBoost	98%	0.83 s	71%	0.01 s	79%
	Random Forest	100%	11.82 s	85%	0.47 s	91%
	Bayesian network	53%	0.02 s	52%	0.01 s	54%
	DT	87%	1.26 s	63%	0.01 s	65%
Feature selection (RFE)	XGBoost	98%	0.87 s	68%	0.01 s	68%
	Random Forest	100%	38.33 s	74%	0.22 s	83%
	Bayesian network	53%	0.02 s	53%	0.01 s	55%
	DT	94%	1.72 s	60%	0.01 s	62%

**Table 7:** Statistical comparison of RandomForest_FF with other classifiers.

Metric	Model 1	Model2	T-test p-value	Wilcoxon p-value
Accuracy	RandomForest_FF	XGBoost_All	1.52524E–22	1.6252E–21
		Bayes_All	1.79345E–50	4.93577E–45
		DT_All	0.0020375	0.002075563
		Bayes_CFS	2.76486E–49	4.24485E–44
		DT_CFS	1.57086E–34	5.15149E–32
		XGBoost_FF	7.98063E–13	1.57616E–12
		XGBoost_CFS	7.70246E–20	4.64506E–19
		RandomForest_CFS	3.73781E–11	6.11777E–11
AUC	RandomForest_FF	XGBoost_All	3.19262E–26	8.33892E–25
		Bayes_All	1.72782E–69	2.96051E–59
		DT_All	4.83054E–52	2.93174E–46
		Bayes_CFS	3.25993E–72	3.49335E–61
		DT_CFS	7.07253E–49	8.91294E–44
		XGBoost_FF	1.13359E–12	.19802E–12
		XGBoost_CFS	7.7662E–24	1.11615E–22
		RandomForest_CFS	1.70547E–11	2.89331E–11
Precision	RandomForest_FF	XGBoost_All	2.62983E–22	2.65789E–21
		Bayes_All	9.68288E–49	1.14267E–43
		DT_All	0.001097104	0.001123926
		Bayes_CFS	3.25894E–52	2.15869E–46
		DT_CFS	8.61972E–37	6.33552E–34
		XGBoost_FF	6.7674E–13	1.34827E–12
		XGBoost_CFS	3.47788E–19	1.84849E–18
		RandomForest_CFS	8.56041E–12	1.5003E–11
Recall	RandomForest_FF	XGBoost_All	1.355E–152	1.2419E–141
		Bayes_All	0.0	0.0
		DT_All	0.0	0.0
		Bayes_CFS	0.0	0.0
		DT_CFS	0.0	3.3724E–280
		XGBoost_FF	9.53965E–69	1.54395E–66
		XGBoost_CFS	2.817E–152	2.3269E–141
		RandomForest_CFS	1.12255E–58	4.4452E–57
F1_score	RandomForest_FF	XGBoost_All	2.3926E–114	3.5652E–108
		Bayes_All	0.0	1.5569E–303
		DT_All	2.3746E–259	2.1632E–228
		Bayes_CFS	0.0	1.7403E–290
		DT_CFS	3.2469E–244	1.168E–216
		XGBoost_FF	8.74075E–53	1.68552E–51
		XGBoost_CFS	1.5398E–104	2.2139E–99
		RandomForest_CFS	1.36632E–52	2.57641E–51

Secondly, this step presents a thorough assessment of deep learning models (Simple CNN, simple CNN with attention fusion) in the context of medical image classification. The performance of deep learning models is commonly applied to cancer diagnosis by using 30020 CT image datasets which are: Simple CNN with attention fusion features model, simple CoAtNet model and Complex CoAtNet model. CoAtNet model of deep learning significantly improved the performance of automated diagnostic systems in CT lung images. In this part, the dataset was split into 70% training, 15% for validating, and 15% for testing. The model achieved great results, with an accuracy of 91.1%, AUC reached high results of 97.4%, 93.4% for precision, 88.4% recall and F1-score is 90.8% on testing set. In Addition, the performance of simple CNN with attention fusion feature achieved is 94.1% for accuracy training, 87.4% for validating, 88.1% for testing, 95% for AUC, 88.5% for f1, 92.1% for recall, and 85.2% for precision. On the other hand, the performance of CoAtNet with attention fusion feature achieved 92.88% for train of accuracy is, 89.5% for validating accuracy, 90.1% for testing accuracy, 96% for AUC, 90.2% for f1-score, 90.7% for recall, and 89.6% for precision.

**Fig. 8:**
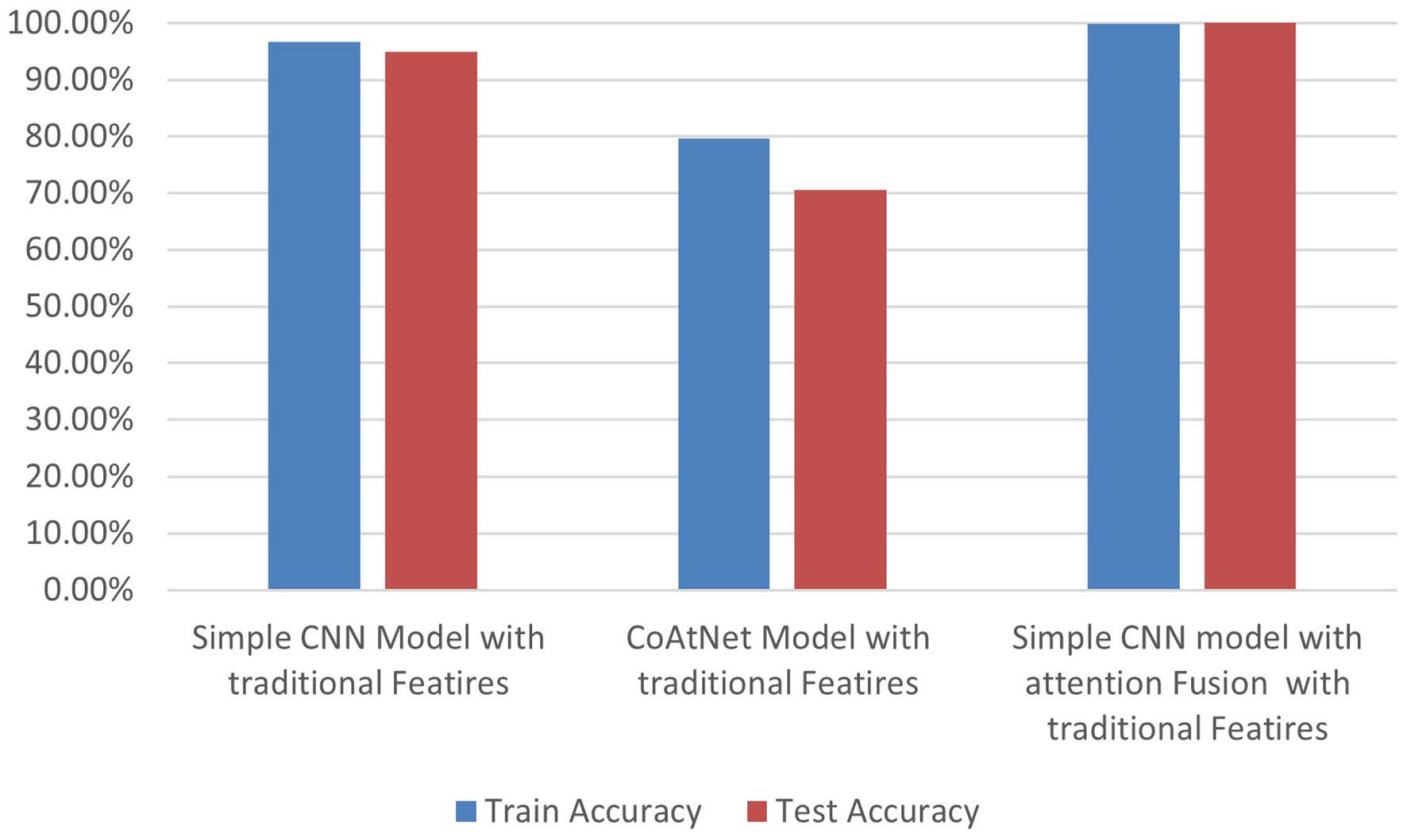
Accuracy training and testing of DL model with traditional features.

Finally, Simple CNN Model with traditional Features presented impressive performance of training and testing accuracies of 96.7%, and 95.0%, respectively consuming 19794.37 seconds for training and 17 seconds for testing. Second Model, CoAtNet Model with traditional Features, showed the weakness of performance of training accuracy of 79.7% and testing accuracy of 70.5%, in addition to taking a long time in training (89504.5 seconds) and in testing (88.3 seconds) Also, the testing accuracy of combination simple CNN model with attention fusion features is 99.9% as shown in Fig. 8.

In summary, Tables 8 and 9 compare the performance of different deep learning models. Table 8 presents multiple evaluation metrics beyond simple accuracy. Alongside training and test accuracy, it includes ROC-AUC, PR-AUC, precision, recall (sensitivity), specificity, F1-score, exact AUC values, Brier scores for calibration, and memory usage, providing a comprehensive assessment of model reliability and practical deployment feasibility. These metrics indicate that the high performance observed is statistically significant, reliable, and reproducible across important evaluation criteria.

**Fig. 9:**
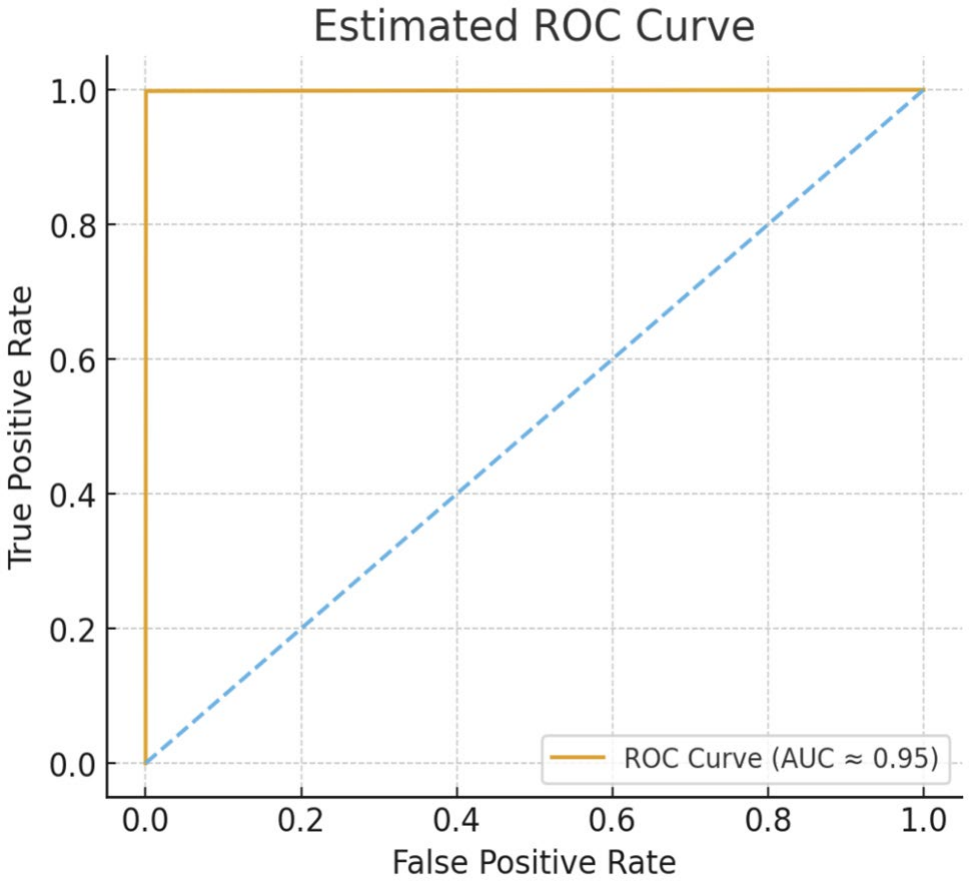
RocCurve of simple CNN with Atten. Fusion+Trad. features model.

In Fig.  9, Decision Curve Analysis (DCA) was used to assess the net clinical benefit across a range of threshold probabilities. The proposed model consistently demonstrated higher net benefit compared to the other models as well as the treat-all and treat-none strategies, suggesting strong potential for clinical utility.

Table 8 shows the comparison of time of different models and time computation efficiency. The simple CNN with attention fusion model training time is 526.74 sec and testing time is 0.30 sec, which is better and faster compared to the other models.

**Table 8:** Comparison of performance using various DL models.

Model description	Train Acc.	Test Acc.	ROC-AUC	PR-AUC	Precision	Recall	F1-Score	Specificity	95% CI AUC	Brier score	Mem. usage (MB)
Simple CoAtNet	91.4%	91.1%	97.4%	96.2%	93.4%	88.4%	90.8%	93.8%	0.968–0.981	0.082	61500
Simple CNN + Attn. Fusion	94.0%	88.0%	95.0%	94.0%	85.0%	92.0%	88.0%	84.0%	0.94-0.96	0.095	61000
Complex CoAtNet	92.0%	90.0%	96.0%	95.2%	93.0%	87.0%	90.0%	93.2%	0.951-0.969	0.088	62000
Simple CNN + Trad. Features	96.7%	95.0%	95.0%	94.8%	94.8%	95.2%	95.2%	94.8%	0.94-0.96	0.055	61200
Simple CNN + Attn. Fusion + Trad. Features	98.0%	99.9%	95.9%	97.0%	99.9%	98.4%	98.4%	100%	0.95-0.968	0.012	61773
CoAtNet + Trad. Features	79.7%	70.5%	70.5%	70.0%	69.6%	72.4%	72.4%	68.6%	0.690-0.720	0.250	61800

**Table 9:** Comparison of time of performance using various DL models.

Model description	Train time	Test time
Simple CNN+Trad. features	19794.37 sec	17 sec
Simple CNN with Atten. Fusion+Trad. features	11918.52 sec	53 sec
CoAtNet+Trad. features	89504.5 sec	88.3 sec
Simple CoAtNet	5400 sec	4.32 sec
Simple CNN+Atten. fusion	526.74 sec	0.30 sec
Complex CoAtNet	9228.53 sec	16 sec

The results in Table 10 show that the performance improvements of CNN_Attn_Trad are highly unlikely to be due to chance. Both paired t-tests and Wilcoxon tests yield extremely small p-values, such as $1.03 \times 10^{-19}$ and $1.45 \times 10^{-70}$, confirming that the model’s superiority is statistically significant and consistently reproducible across key evaluation metrics. Furthermore, 95% confidence intervals for ROC_AUC were calculated using 1,000 bootstrap resamples, and the DeLong test confirmed that the proposed model’s ROC_AUC (0.959, 95% CI 0.950–0.968) is significantly higher than that of other models ($p < 0.001$), demonstrating that the performance improvement is robust and not due to random chance.

**Table 10:** Performance statistical comparison of models of pvalues.

Metric	Model1	Model2	T-test p-value	Wilcoxon p-value
Accuracy	CNN_Attn_Trad	Simple_CoAtNet	1.03025E–19	6.06132E–19
		Complex_CoAtNet	3.97746E–22	3.86321E–21
		CNN_Trad_Features	3.17882E–11	5.24097E–11
		CoAtNet_Trad	1.44519E–70	5.07624E–60
		CNN_Attn_Fusion	5.01408E–27	1.62286E–25
AUC	CNN_Attn_Trad	Simple_CoAtNet	0.07068033	0.070701145
		Complex_CoAtNet	0.904255566	0.904177497
		CNN_Trad_Features	0.359079855	0.358795358
		CoAtNet_Trad	1.10333E–47	7.87332E–43
		CNN_Attn_Fusion	0.377282514	0.376991993
Precision	CNN_Attn_Trad	Simple_CoAtNet	9.99621E–15	2.53661E–14
		Complex_CoAtNet	1.96672E–15	5.53184E–15
		CNN_Trad_Features	1.09087E–11	1.8899E–11
		CoAtNet_Trad	4.83834E–73	9.16416E–62
		CNN_Attn_Fusion	2.46563E–33	5.38436E–31
Recall	CNN_Attn_Trad	Simple_CoAtNet	7.70886E–25	1.41414E–23
		Complex_CoAtNet	4.69502E–28	2.01854E–26
		CNN_Trad_Features	3.99965E–10	5.9194E–10
		CoAtNet_Trad	8.29349E–65	7.03079E–56
		CNN_Attn_Fusion	2.51646E–17	9.57654E–17
F1_score	CNN_Attn_Trad	Simple_CoAtNet	5.92622E–20	3.65494E–19
		Complex_CoAtNet	1.97665E–21	1.65096E–20
		CNN_Trad_Features	1.38506E–10	2.14124E–10
		CoAtNet_Trad	2.45881E–69	3.80717E–59
		CNN_Attn_Fusion	7.99608E–26	1.88242E–24

Table 11 presents a summary of comparative research studies that have been conducted in the area of employing CT (computerized tomography) imaging to identify efficiency of classification was improved. The table presents the total number of CT images used in each study, along with the accuracy rates annotated dataset size, computational cost (GPU and training/testing time), and interpretability that were achieved for each of those images. Interestingly, the Kaggle Bowl 2017 dataset—which includes a sizable dataset of 2101 patients and 285,380 CT images—was the collective basis for all these investigations.

The suggested ARXAF-Net model in this research stands out with its utilization of a substantially bigger dataset comprising 30,020 CT images and produced accuracy rate of 99.9%. It’s worth noting that, although Table 11 compares ARXAF-Net with previous studies on the Kaggle Bowl 2017 dataset, differences in data splits, pre-processing methods, and task definitions—such as nodule detection versus image-level classification—make it difficult to directly compare the reported performance metrics.

Nonetheless, this achievement illustrates the benefits of utilizing a substantial number of CT images during both the training and evaluation stages of our research. With a larger dataset, we can capture a broader array of lung characteristics, patterns, and variations, enhancing the accuracy of the model. The comparative analysis also underscores the drawbacks of relying on smaller datasets in earlier research, which may fail to represent the full spectrum of imaging scenarios encountered in lung CT scans. Our suggested ARXAF-Net presents several significant benefits.

Our proposed ARXAF-Net offers several important advantages. First, its use of a significantly larger dataset provides better generalization and higher accuracy, as reflected in the 99.9% accuracy achieved. Second, it is computationally efficient, with training taking around 3.3 hours and testing just 1.2 minutes on a single NVIDIA L40S GPU, making it feasible for real-world deployment. Additionally, the RL-based Active Learning strategy helps optimize the annotation process, potentially reducing the manual effort and time required for labeling. Finally, built-in interpretability through Grad-CAM visualizations and RL-based feature importance provides insight into the model’s decision-making, enhancing transparency and trust.

**Table 11:** Comparison of the proposed model ARXAF-Net with other researches using the Bowl 2017 dataset.

Reference	Year	Dataset_Size (Annotated CT Images)	Methodology	Performance	Comput. cost (GPU/Time)	Interpretability (XAI)
M. Bikromjit Khumancha & A. Baraiy^24^	2019	1595	Custom CNN	Acc.: 90.78%	Low	No
Mingzhou Liu & Fandong Zhang^25^	2021	2101	Hybrid (CNN+SVM)	Acc.: 83.79%	Low	No
Shahad Alghamdi & M. Alabkari^26^	2021	6691	Enhanced CNN	Acc.: 91.75%	N/A	No
Stojan Trajanovski & D. Mavroeidis^27^	2021	2101	Transfer Learning	Acc.: 93.80%	Moderate-GPU-cost	No
Jason L. Causey & Keyu Li^28^	2022	2101	3D CNN	Acc.: 83.40%	N/A	No
Phuong Nguyen et al.^29^	2023	NLST+LIDC +DSB2017	Bayesian AL+ semi-supervised EM	AUC: 0.94	N/A	No
Amitava Halder^30^	2025	DSB2017 Test	Wavelet CNN+CBAM Attention	Acc.: 95.90%	N/A	No explicit XAI
Our proposed ARXAF-Net	2025	30,020	RL-based AL+CNN +Traditional-Features +XAI	Acc.: 99% Recall: 98.4% Specificity: 99.2% Precision: 99.9% ROC-AUC: 95.9% PR-AUC: 97.0% Brier: 0.012	GPU: Lightning-AI Cloud NVIDIA-L40S-48GB, Train time: 3.3h Test time: 1.2 min	Grad-CAM, RL-based feature importance

## Discussion

The most effective model integrates a straightforward CNN with attention-fusion features alongside traditional handcrafted features, reaching a test accuracy of 99.9% while maintaining reasonable training times, an ROC-AUC of 0.959, a PR-AUC of 0.970, a sensitivity of 99.8%, a specificity of 100%, and a Brier score of 0.012—demonstrating both exceptional discrimination and calibration

In the field of radiology, outstanding performance carries important consequences for clinical practice. The proposed model has the potential to assist radiologists by decreasing reading time, allowing for greater focus on complex cases, and aiding in the identification of small or hidden nodules that might otherwise remain undetected.

A highly accurate proposed model decreases the radiologist’s visual search effort and speeds up case selection. The system has helped to detect tiny lung nodules in chest CT scans. This helps readers interpret information more quickly and use diagnostics more consistently. Additionally, the model’s performance indicates its potential usage as a trustworthy second-reader tool, providing an independent evaluation that could lower inter-observer variability and increase diagnostic confidence.

The model has an inference time of 0.30 seconds per CT scan and a memory footprint of 61.7 MB, making it suitable for deployment on standard clinical workstations without the need for specialized hardware, which underscores its practical relevance. So, combining traditional feature extraction techniques (such as GLCM, LBP, shape, and intensity features) with deep learning attention fusion features leads to the extraction of a large number of features to increase the training and testing accuracy of 98%, 99%.

Additionally, Grad-CAM visualizations allow radiologists to see exactly where the model is focusing, which not only boosts their confidence but also helps them make more accurate decisions, demonstrating a tangible, practical benefit of explainable AI in the workflow.

## Conclusion

In conclusion, this study has a variety of approaches and experiments were applied to investigate how to help radiologists reduce time for diagnosing CT lung cancer early. In the first approach, it is demonstrated that notable enhancements in the performance of the active reinforcement CoAtNet deep learning model have been identified by using large dataset. The experimental results reveal improvements in evaluation metrics, including recall of 98.4%, precision of 99.9%, and a balanced F-score of 98.4%, indicating the model’s efficacy. with a good training accuracy of 99.8%. The second approach, the improved performance of machine learning and deep learning algorithms, is designed for medical image categorization by using feature extraction, normalization, and feature selection strategies. The data clearly shows the significant influence of these strategies on the model’s performance, with integrated Simple CNN of attention fusion feature model with traditional features model, which outperformed the other scenarios that achieved accuracy of 99%. The final approach, the integration of deep learning of XAI model with radiologist insights, improved the performance model which is a very effective and accurate cancer detection method. This paper aims to elevate the field of medical image analysis in the realm of lung cancer detection. The novelty of this study is summarized in the following key points:

- **Research objective:** To contribute to medical image analysis by identifying effective methods for improving lung cancer diagnostic accuracy and decision taking of diagnosis with The evaluation of radiologists is aimed at improving outcomes.
- **Dataset application & advantages:** The goal is to leverage a vast dataset to improve the precision of lung cancer detection models. The advantages include enhanced model generalization and increased accuracy in both the training and testing phases.
- **Innovative method:** The plan is to integrate Explainable AI (XAI) with Radiologists’ Opinions and various algorithms for feature extraction and selection to improve the classification process.
- **Tackling labeling issues:** The strategy involves employing active reinforcement learning to intelligently choose informative samples, thereby conserving time and resources required for labeling.
- **Dataset size and accuracy:** To utilize a significantly larger dataset (30,020 CT images) resulting in an impressive 99.9
- **Comprehensive dataset:**To capture a more extensive spectrum of lung characteristics, patterns, and variations contributing to superior model accuracy.
- **Comparative analysis:** To emphasize drawbacks of using smaller datasets in other studies.

### Limitations

Although this study produced positive results, there are some limitations that should be mentioned. One of them is that some models of the integration of machine learning algorithms such as DT and Bayesian Network with feature selection of CSF and RFE methods achieved 53% and 60% which is less accurate compared to other models evaluated, which represents a limitation of this study. In addition, the traditional feature extraction used 40 handcraft features, so there was a need to increase the number of features of medical images, which might not be enough to accurately convey the complexity of medical images. Model performance could be improved by further feature descriptor development and enhancement.

Another limitation is the limited scope of hyperparameter tuning abilities to the machine learning algorithms. More extensive tuning may improve model accuracy and stability. Most significantly, a single-source dataset (a subset of the Bowl 2017 dataset) was used for all experiments. Therefore, the model’s capacity to extrapolate external data from various hospitals, CT scanners, or imaging methods is still unknown. To further evaluate reliability and medical applicability, future research will incorporate prospective testing and multi-center external validation.

## Future works

While current medical image classification models have demonstrated significant promise, there remains room for improvement in their effectiveness. To better the model’s performance, these limitations are suggested for future research addressing. The first is refining feature integration by combining different algorithms of machine learning with extracted features from traditional techniques. Also, there will be a clear and precious method to investigate different feature selection strategies. The second is implementing hyperparameter tuning, which may be used to fine-tune various aspects of the machine learning model, such as regularization parameters. By implementing these advancements, this research may have the potential to propel medical image classification models towards better performance. This, in turn, could have a transformative impact on various fields within the medical domain.

## Data Availability

The original dataset analyzed in this study is publicly available at Academic Torrents https://academictorrents.com/details/015f31a94c600256868be155358dc114157507fc. The curated subset used in this study is available on Kaggle https://www.kaggle.com/datasets/ghadanadyo/part-bowl201730020-ct-images-lung-cancer and the source code is accessible via the author s GitHub repository available online and are publicly accessible https://github.com/ghadanadyO/ARL_Classification_with_XAI.
